# Synthesis, characterization and application of a zirconium-based MOF-808 functionalized with isonicotinic acid for fast and efficient solid phase extraction of uranium(VI) from wastewater prior to its spectrophotometric determination

**DOI:** 10.1186/s13065-022-00821-1

**Published:** 2022-04-16

**Authors:** Marzieh Sharifi-Rad, Massoud Kaykhaii, Mostafa Khajeh, Alireza Oveisi

**Affiliations:** 1 Department of Chemistry, Faculty of Sciences, University of Sistan and Baluchestan, Zahedan, Iran; 2 Department of Process Engineering and Chemical Technology, Faculty of Chemistry, Gdansk University of Technology, Gdansk, Poland; 3 Department of Chemistry, University of Zabol, Zabol, Iran

**Keywords:** Uranium, Metal organic framework, Sample preparation, MOF-808, Spectrophotometry, Wastewater analysis

## Abstract

**Background:**

A zirconium-based metal-organic framework (Zr-MOF), named MOF-808, was synthesized and fully characterized by solvo-thermal method and functionalized by isonicotinic acid and employed as an efficient adsorbent for selective extraction and preconcentration of uranyl ions from water and waste water samples in a batch solid phase extraction.

**Results:**

Parameters affecting extraction such as volume and pH of the sample solution, the amount of sorbent, type and volume of eluting solvent, and adsorption and desorption times were investigated and optimized. Under the optimized conditions, high extraction efficiency was observed with a limit of detection of 0.9 µg L^− 1^ for uranyl ions and relative standard deviations were found to be better than 2.1% in the range of 0.07–1000 µg L^− 1^.

**Conclusions:**

These results indicated that the above procedure is fast, inexpensive, effective, reliable, applicable and organic solvent-free and showed the highly performance and stability of the Zr-MOF in SPE based analytical techniques.

## Introduction

Uranium is a heavy metal element that can be naturally found at low levels in all parts of the environment [1]. It can be entered to water because of mining, test of nuclear weapons and leaking from nuclear facilities. Groundwater contamination with uranyl ions in different parts of the world is extensively reported [2–5]. Entrance of this element to human body is dangerous because of both its chemical toxicity and its radioactivity. While uranium is dangerous to the most of body organs, including brain, liver, and heart, its main effect is on kidneys. Daily intake of only small amounts of U in tap water causes brain damage because of its deposition in hypothalamus. Uranium also increases the risk of cancer in human [6]. According to the World Health Organization, maximum level of uranium in drinking water should not exceed 15 µg L^− 1^ [7]. As a result, monitoring of this element is a requirement in many environmental monitoring programs. So far, several instrumental methods such as ion chromatography [8], inductively coupled plasma atomic emission spectrometry [9], capillary zone electrophoresis [10] and inductively coupled plasma-mass spectrometry [11] have been developed for determination of uranyl ions, but they are very expensive for routine use. Other techniques such as liquid scintillation [12], laser fluorimetry [13] and alpha spectrometry [14] and gamma spectrometry [15] cannot be used in small laboratories or for on-site monitoring. However, ultraviolet-visible spectrophotometry (UV-Vis) is a simple and low cost technique with appropriate precision and accuracy, which is available in the most of laboratories and can be used for the determination of U after its complexation with selective ligands. The critical point against the use of spectrophotometry for determination of uranium is associated with its low sensitivity and impossibility of direct determination without sample preparation, because it is not sensitive to low concentration of the uranyl ions [16].

Solid phase extraction (SPE) is a very popular technique currently in use for the separation and preconcentration of trace elements in almost all kind of samples, due to the advantages such as ease of use, relatively low cost, adequate analyte recovery, high selectivity, high speed, less consumption of organic solvents, and ability to be automated, [17]. It has been applied as a very efficient technique for the extraction and preconcentration of uranium and thorium from environmental samples [18], extraction of radionuclides from aqueous media [19], and as automatic methodology for thorium(IV) and U(VI) determination in seawater samples [20].

Metal organic frameworks (MOFs) are a class of crystalline hybrid materials that metal ions or metal clusters can be linked by various organic bridging ligands [21, 22]. The highly porous structure of MOFs provides this ability that guest species such as metal ions diffuse into the bulk structure and be selectively absorbed by size and shape of pores. Because of these features, MOFs are ideal sorbents for solid phase extraction of heavy metals [23]. In this regards, Zr-based metal organic frameworks (Zr-MOFs) are one of the best MOFs because of their excellent thermal, mechanical, and chemical stability [24, 25]. Since they contain a structure which has water-tolerant across a wide range of pHs, they can be used in many applications which need to be performed in both acidic and basic media [26]. MOF-808 is a type of Zr-MOF that is assembled from Zr_6_(µ_3_-O)4(µ_3_-OH)_4_ nodes connected to both six 1,3,5-benzenetricarboxylate (BTC^3−^) linkers and charge-balancing formate ligands. Nanocrystals of this MOF can be synthesized by solvothermal method under elevated temperatures and long reaction times [27].

In this paper, a simple and accurate spectrophotometric method for determination of trace amounts of uranyl ions in wastewater samples is developed based on SPE of the U(IV)-arsenazo (III) complex, using Zr-MOF functionalized by isonicotinic acid (INA) as a selective, high capacity sorbent. The reaction of uranyl hydroxyl with INA forms (UO_2_)(OH)(INA) complex. It has a 2D layered structure that links INA anions pillars of the uranyl hydroxyl columns to the U(VI) compound. U(VI) compound with INA is formed in µ3-bridging mode via bridging carboxylate group and U–N bonding. At pHs higher than 5.4, further formation of this complex occurs by hydrolysis of uranyl species. Raman spectroscopic investigating show significant red-shift due to the effect of the pentagonal pyramidal structure of the INA anion and the bridging carboxylate group and U–N bonding [28]. The experimental conditions of extraction were studied and optimized.

## Experimental

### Reagents

Analytical grade reagents, uranyl acetate dihydrate (UO_2_(CH_3_COO)_2_.2H_2_O), zirconium tetrachloride (ZrCl_4_), trimesic acid (BTC), glacial acetic acid (CH_3_COOH), trichloromethane (CHCl_3_), acetone (C_3_H_6_O), hydrochloric acid (HCl), methanol (CH_3_OH), isonicotinic acid (C_6_H_5_NO_2_), acetonitrile (C_2_H_3_N), N,N′-dimethyl formamide (DMF) and Arsenazo (III) were purchased from Sigma-Aldrich (MO, USA) without further purification. A stock standard solution of uranium (1000 mg L^− 1^) was prepared by dissolving uranyl acetate dihydrate in deionized water and daily solutions were prepared by its proper dilution. 1000 mg L^− 1^ of Arsenazo (III) was prepared in deionized water as a stock solution and used as ligand after proper dilution.

### Apparatus

Absorption measurements at 652 nm wavelength (analytical wavelength of the uranyl-arzenazo complex) were performed by a 2100 RAYLeigh (Beijing, China UV-Vis) double-beam spectrophotometer. Fourier transform infrared (FTIR) spectra were recorded by a version 10.01.00 Perkin-Elmer instrument (model Spectrum (USA)) in the range of 4000–500 cm^− 1^ using KBr pellets. For characterization and investigation of morphology and chemical composition of the synthesized INA@MOF-808 MOF, the following instruments were used. A scanning electron microscope (SEM) equipped with energy dispersive X-ray (EDX) model MIRA3 (TESCAN, Czech Republic) was utilized to take images. A Philips X’pert (the Netherlands) powder X-ray diffraction (PXRD) diffractometer at Cu K_α_ radiation (λ = 1.5418 Å, 293 K) was employed for obtaining patterns within the range of 1.5°<2θ < 50°. Thermogravimetric analyses (TGA, Mettler Toledo, Swiss) were performed under nitrogen atmosphere, for which, samples were heated from room temperature to 700 °C at 10 °C.min^− 1^. N_2_ adsorption-desorption isotherms were measured at 77 K on a Micromeritics TriStar II 3020 (Norcross, GA, USA) porosity and surface area analyzer. TriStar II 3020 V1.03 software (Micromeritics) was used for data analysis. pHs of solutions were measured by a model 630 Metrohm pH meter (Swiss) with glass electrode. All experiments were repeated at least three times and the mean values were used.

### Synthesis of functionalized MOF-808

**Fig. 1:**
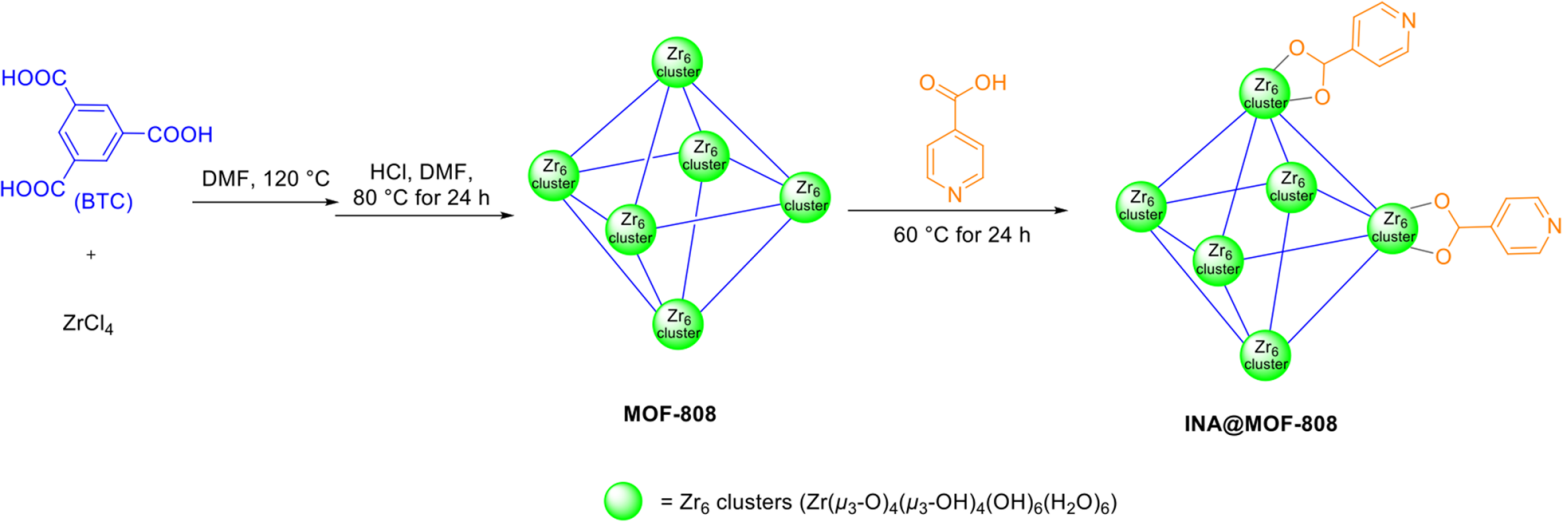
Schematic synthesis of zirconium-based MOF-808 functionalized with isonicotinic acid

Zr-based MOF-808 (MOF-808-Ac, Zr_6_O_4_(OH)_4_(BTC)_2_(CH_3_COO)_6_) was synthesis based on the Jiong et al. method [27]. For its functionalization with isonicotinic acid, 250 mg of the obtained MOF-808 was placed in a screw-capped vial and 55 mL of DMF and 5 mL of concentrated HCl were subsequently added. The suspension was then incubated at 80 °C for 24 h. After it was cooled at room temperature to 30 °C, the solid was collected by suction filtration and extracted overnight by methanol in a Soxhlet extractor. The resulting white precipitate was dried at 100 °C in a vacuum drying oven to obtain MOF-808. This activated MOF-808 was added to 12 mL of 0.05 M solution of isonicotinic acid in dimethyl formamide and kept warm at 60 °C for 24 h. After centrifugation, the supernatant was discarded and the remaining solid was soaked in 12 mL acetonitrile at 60 °C for 24 h to remove unbound ligands from the functionalized MOF. Finally, the resulting materials described here as INA@MOF-808 were washed three times with acetone (3 × 10 mL) and dried at 70 °C for 48 h. A schematic illustration of the mechanism and formation of zirconium-based MOF-808 functionalized with isonicotinic acid is shown in Fig. 1.

### SPE Procedure

All adsorption experiments were performed in 50.0 mL canonical bottom glass vials. 25 mL of sample solution containing uranyl ions was added to the vial and its pH was adjusted to 6.0 by addition of either 0.1 mol.L^− 1^ NaOH or 0.1 mol.L^− 1^ HCl. Then 10 mg of INA@MOF-808 was added and sonicated for 10 min to extract analyte ions. After that, the suspension was centrifuged for 5 min at 7500 rpm (6000 rcf) to separate the adsorbent. Supernatant solution was discarded and 1 mL concentrated HNO_3_ was added as elusion solvent, followed by 15 min sonication. After centrifuging for 5 min at 7500 rpm (6000 rcf), the supernatant was collected by filtration. 1 mL of 25 mg L^− 1^ Arsenazo (III) was added and after 10 min, the concentration of the analyte was determined by UV–Vis spectrophotometer.

The extraction recovery (ER) was obtained by using Eq. (1):

1 $${\text{ER}}\left( \% \right) = \left( {{{\text{C}}_0} - {{\text{C}}_{\text{f}}}/{{\text{C}}_0}} \right) \times 100$$

where C_0_ and C_f_ are the concentrations of uranyl ion before and after extraction.

## Results and discussion

### Characterizations of INA@MOF-808

**Fig. 2:**
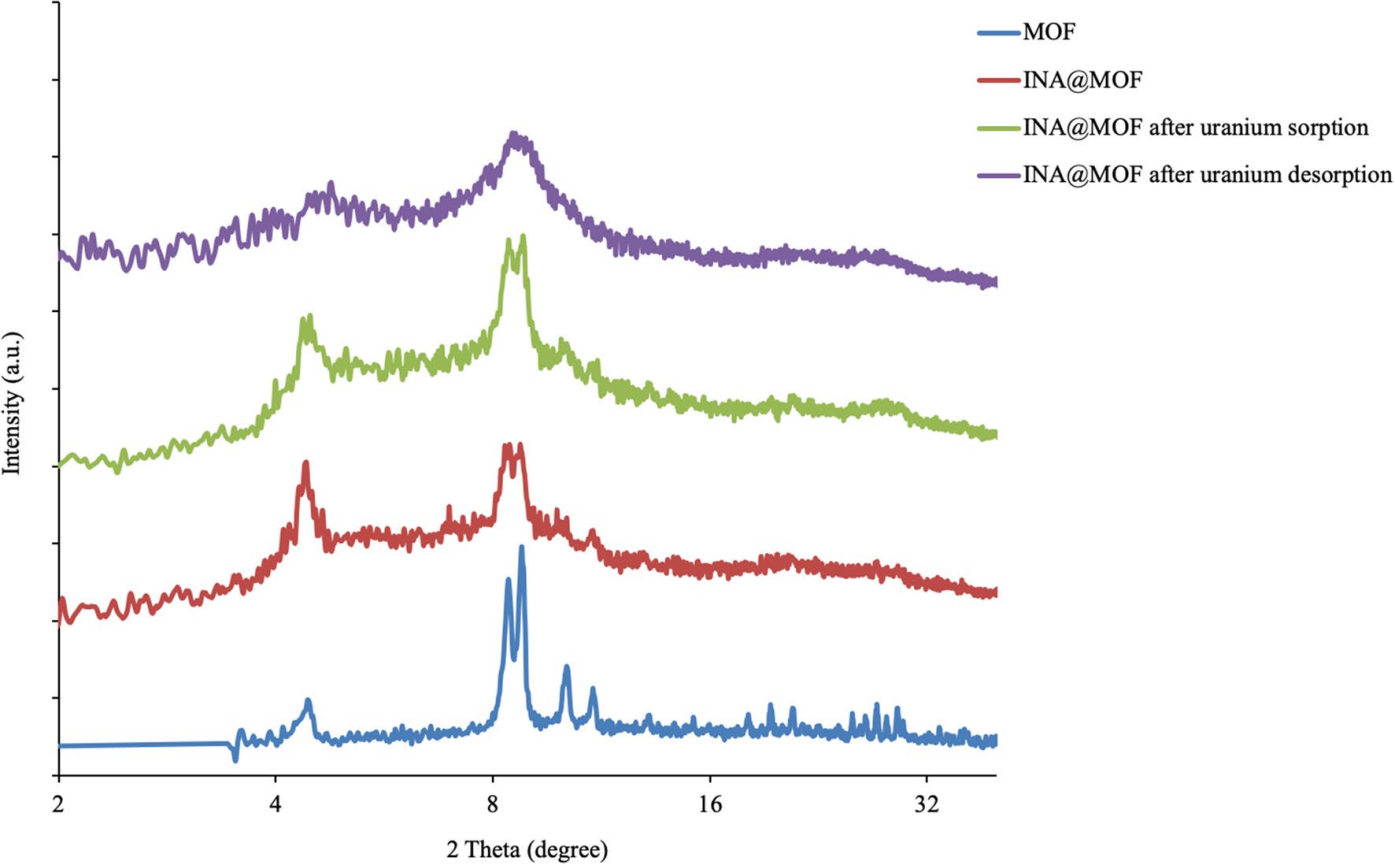
Powder X-ray diffraction patterns of the MOF; MOF-808, INA@MOF-808, INA@MOF-808 before and after analyte desorption for 8 times

The PXRD pattern of synthesized MOF after and before functionalization by isonicotinic acid is depicted in Fig. 2, which indicates a full set of reflections from the crystalline phases of MOF-808 [29]. At 2θ = 4.34°, the diffraction peak can be assigned to the (111) plane of them. The two most intensive peaks at 2θ = 8.32° and 8.69° are respectively related to the (311) and the (222) crystal planes. It can be observed that the peaks are well consistent with what previously was reported [30]. There is no visible difference peak intensity between the crystallinity of MOF-808 and INA@MOF-808 and the MOF maintained its crystallinity in the composite. Besides, PXRD patterns of the Zr-MOF before and after 8 times uranyl ions adsorption are showed in Fig. 2 which confirms that the crystallinity of the MOF is reserved during the experimental conditions, and verifying the stability of the INA@MOF-808.

**Fig. 3:**
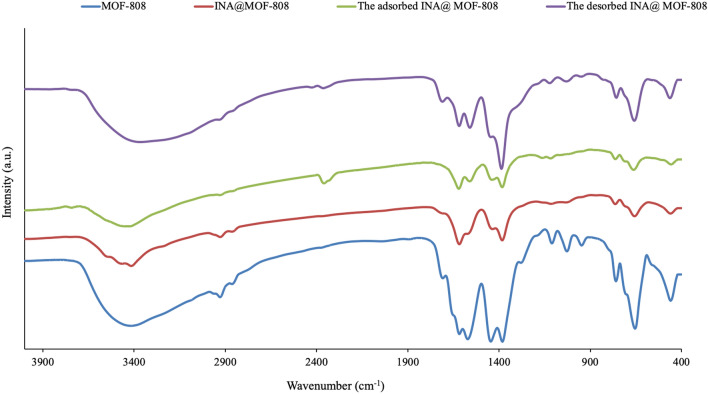
Fourier transformed infrared spectra of the MOF; MOF-808, INA@MOF-808, INA@MOF-808 before and after analyte desorption for 8 times

The FTIR spectrum of the Zr-MOF is showed in Fig. 3. The MOF-808 exhibits the vibration peaks of aromatic rings but without the evident vibration peak of carboxyl group, which indicates that carboxyl groups on all BTC molecules were completely reacted. A broad absorption peak at 3416 cm^− 1^ can be recognized in this spectrum which is related to the symmetric and asymmetric N–H and O–H stretching modes. The intense doublet at 1571 and 1381 cm^− 1^ can be assigned to the symmetrical and asymmetrical stretching modes of the carboxylate groups. The strong vibration peak of Zr-O can be observed at around 650 cm^− 1^, showing that the coordination reaction between the carboxyl in groups of BTC and zirconium ion occurred.

**Fig. 4:**
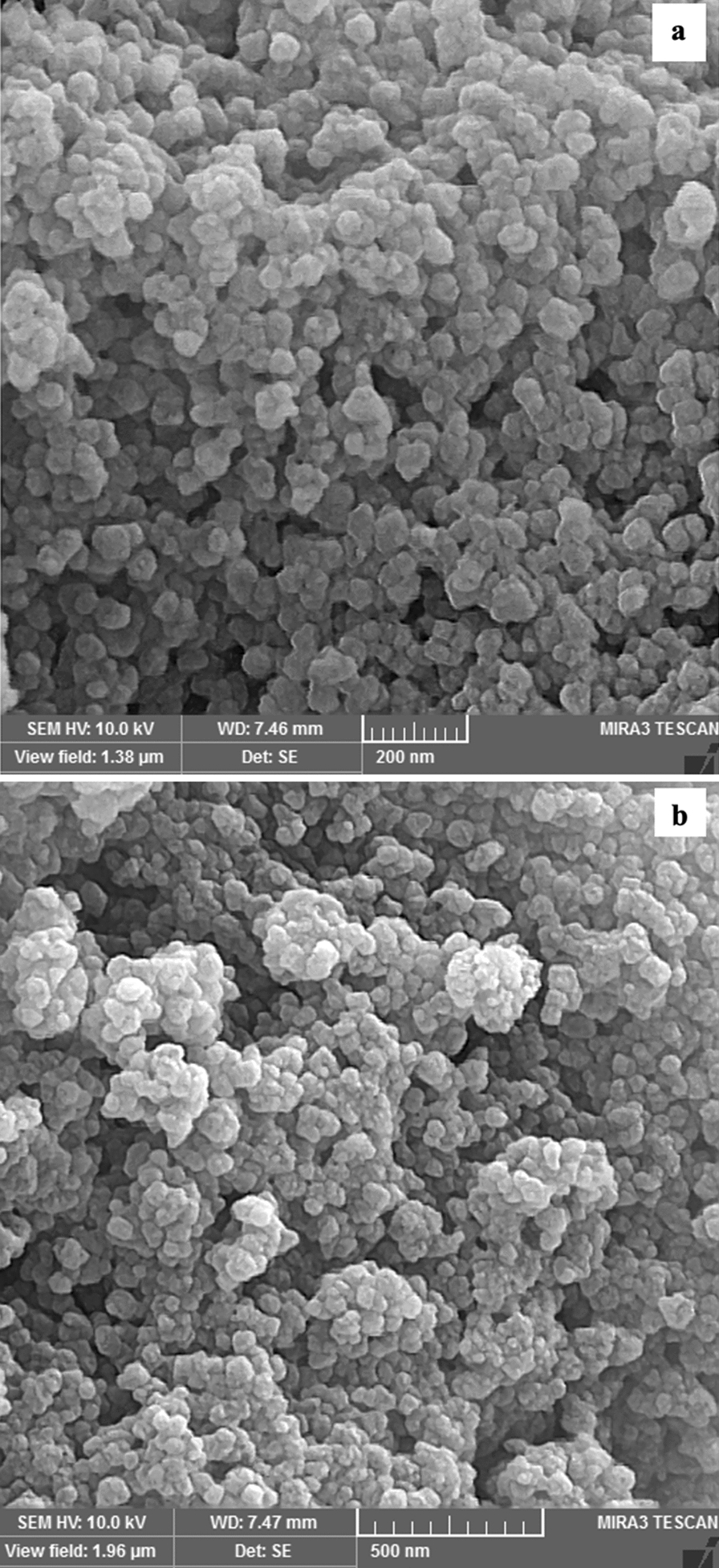
Scanning electron microscope images of INA@MOF-808 with different magnification

The morphological structure of the INA@MOF-808 was investigated by SEM (Fig. 4) which revealed the presence of crystalline shapes with a diameter of 200–700 nm.

**Fig. 5:**
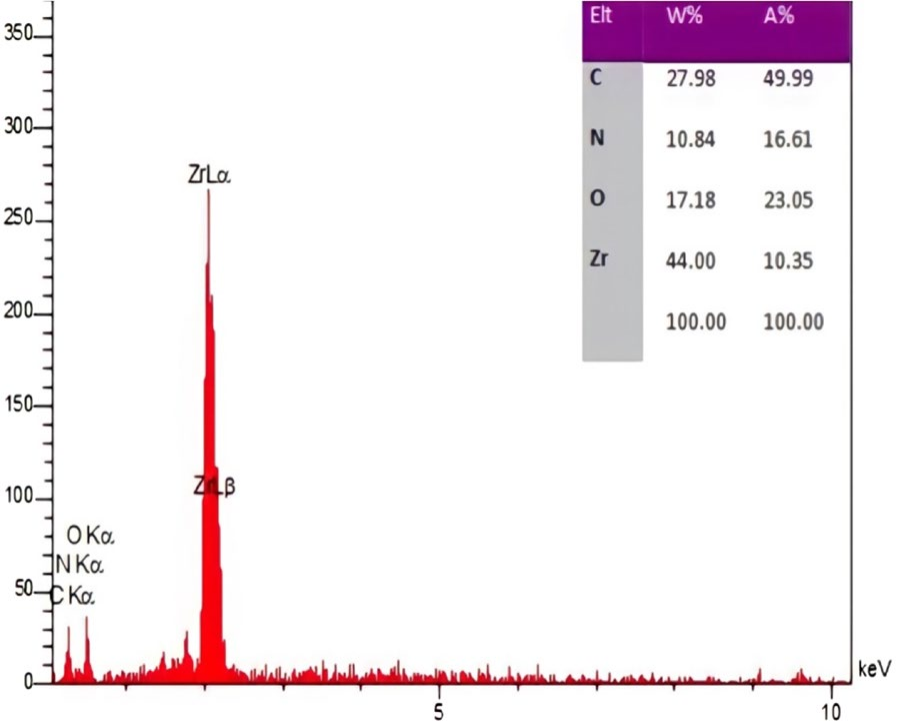
Energy dispersive X-ray elemental analysis of INA@MOF-808

**Fig. 6:**
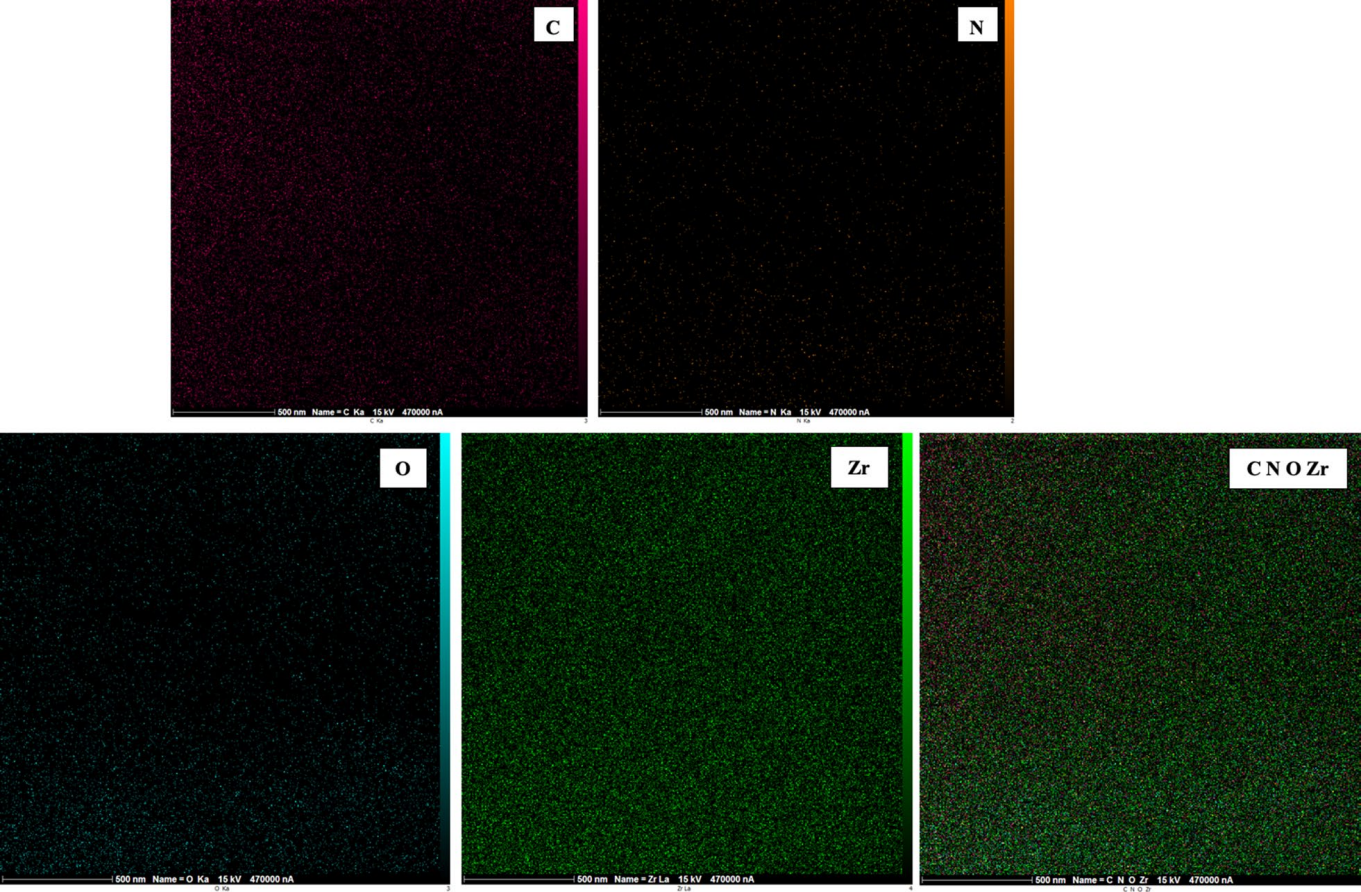
Energy dispersive X-ray elemental mapping of the INA@MOF-808 composite

EDX elemental analysis (Fig. 5) was used for the investigation of the elemental composition of INA@MOF-808 which exhibits the existence of C, O, N and Zr elements, again confirming the successful synthesis of the composite. EDX elemental mapping of the composite depicted that all elements were uniformly distributed in the entire structure (Fig. 6).

**Fig. 7:**
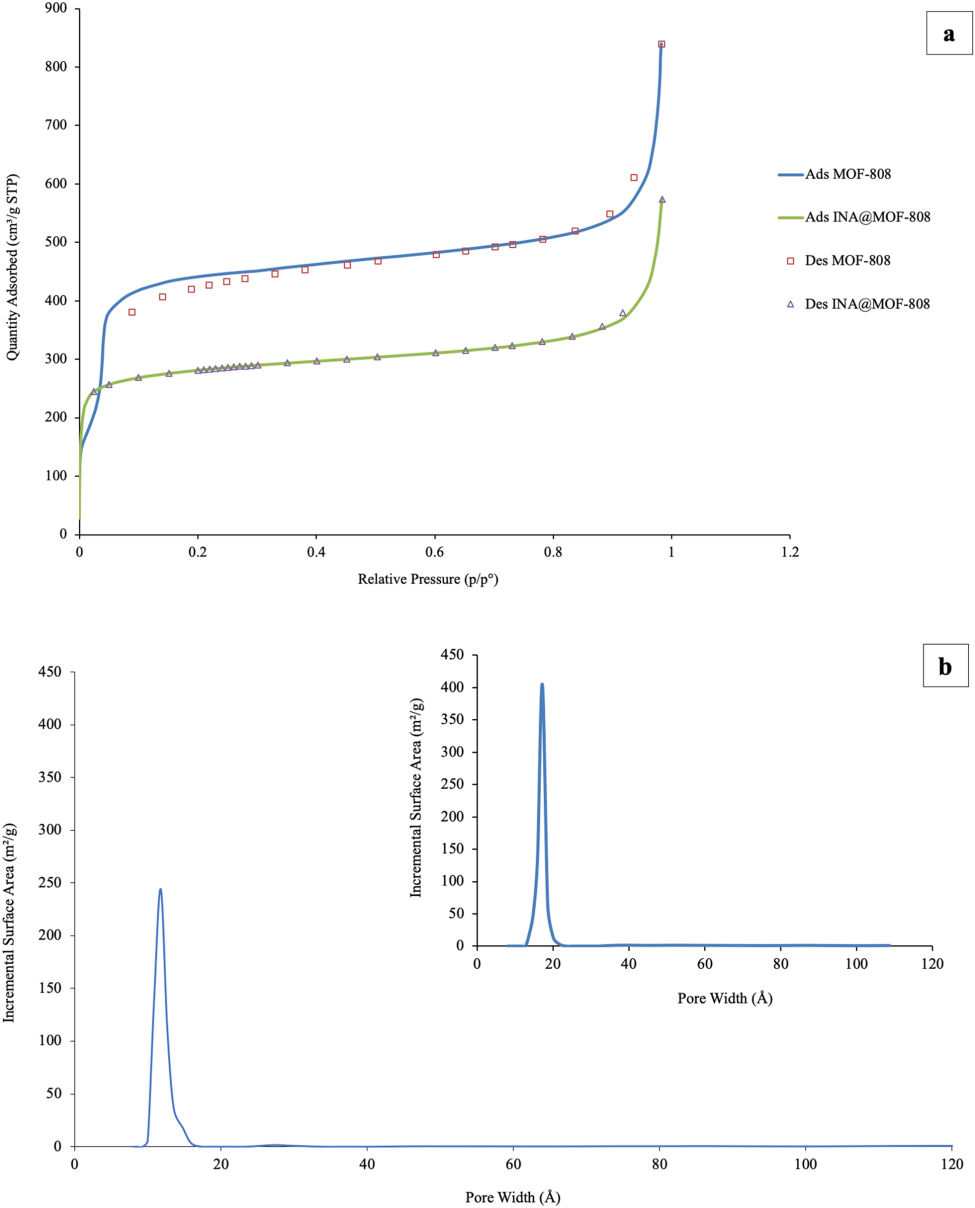
N_2_ adsorption–desorption of MOF-808 and INA@MOF-808 at 77 K (**a**) and pore size distribution of INA@MOF-808 calculated using DFT Model compared with MOF-808 (showed as insert) (**b**)

Brunauer-Emmett-Teller (BET) based on nitrogen adsorption-desorption measurements indicated that INA@MOF-808 is prominently porous with a pore volume of 0.8 cm^3^.g^− 1^ (Fig. 7a). The total surface area was reduced from 1,611.7278 m^2^ g^− 1^ for MOF-808 to 1,075.8769 m^2^ g^− 1^ for INA@MOF-808 after functionalization. Obviously, this reduction caused by the presence of the INA which has a low BET. Furthermore, the isotherm curve of the INA@MOF-808 is typically of type I shape which is representative of a microporous material. Porosity distribution calculated from nitrogen sorption data by density-functional theory (DFT) model confirmed the presence of 1.00–1.17 nm micropores in the framework (Fig. 7b).

**Fig. 8:**
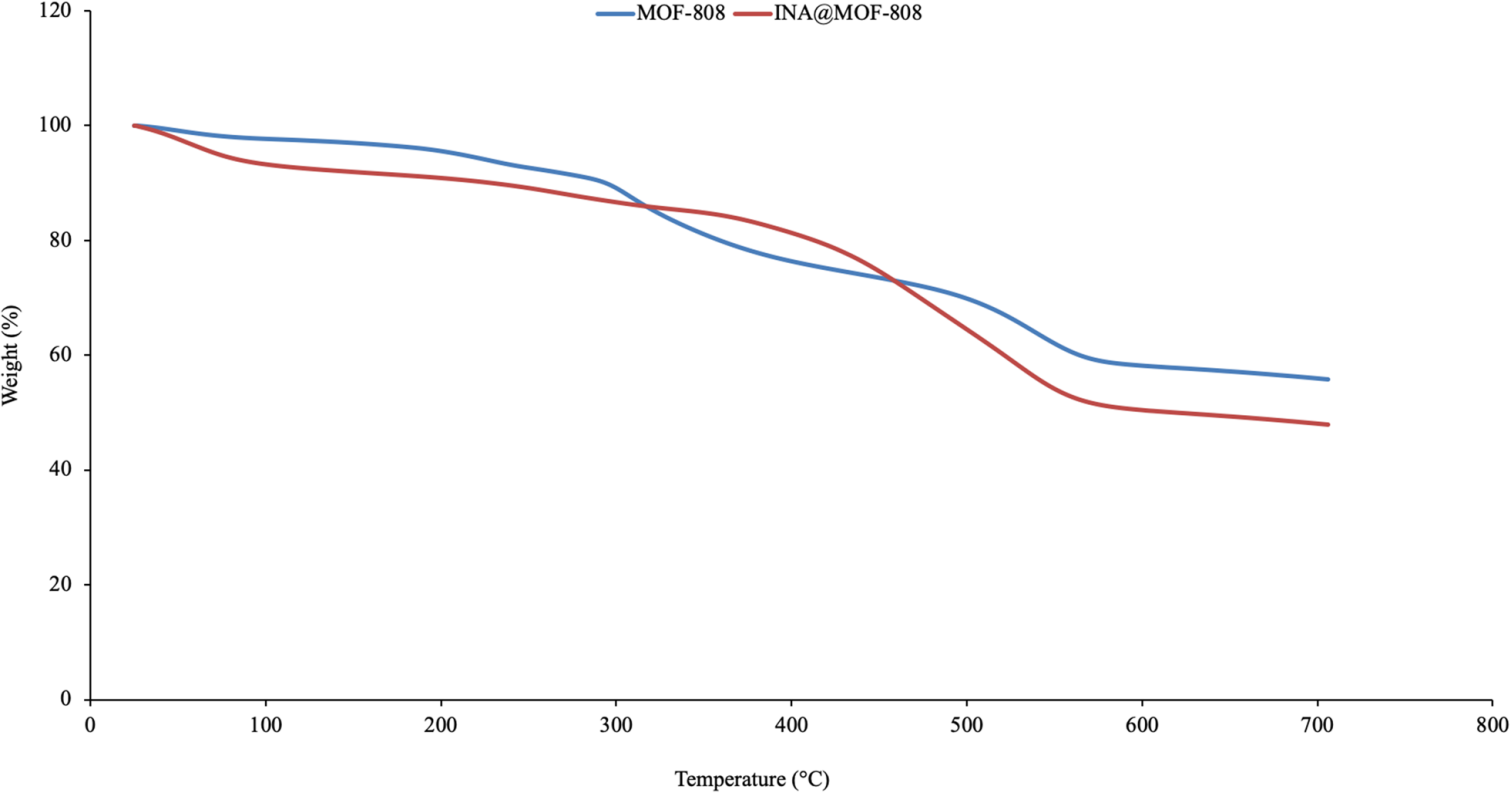
Thermogravimetric Analysis curves of MOF-808 and INA@MOF-808

TGA was employed to evaluate the thermal stability of the synthesized MOFs. TGA curves of MOF-808 and INA@MOF-808 are presented in Fig. 8. TGA curve of INA@MOF-808 showed three significant weight loss stages: first, a 5 wt% loss at 100–200 °C, which is due to the evaporation of the water adsorbed from the environment; a second loss at ca. 200–300 °C originating from the loss of physically adsorbed trimesic acid, and the third loss which is at ca. 300–550 °C caused by the loss of chemisorbed trimesic acid and the breakage of zirconium oxygen coordination bond. Clearly, the synthesized INA@MOF-808 remains stable below 300 °C, which is in agreement with annealed data.

## Optimization of SPE procedure

In order to achieve the best SPE extraction efficiency, parameters which could potentially have an effect on this process were studied and optimized; i.e. pH value of the sample solution, amount of MOF adsorbent, type and volume of the eluent, extraction and desorption time, sample volume and interfering ions. One-parameter-at-a-time method was practiced to optimize the effect of these parameters on the extraction recovery (i.e. the presence of interaction between the factors of the process was not considered), which is widely in use in SPE-based extractions using a MOF [31, 32]. 25.0 mL of 100.0 mg L^− 1^ of uranyl ion standard solution was employed during optimizations.

### Effect of pH

**Fig. 9:**
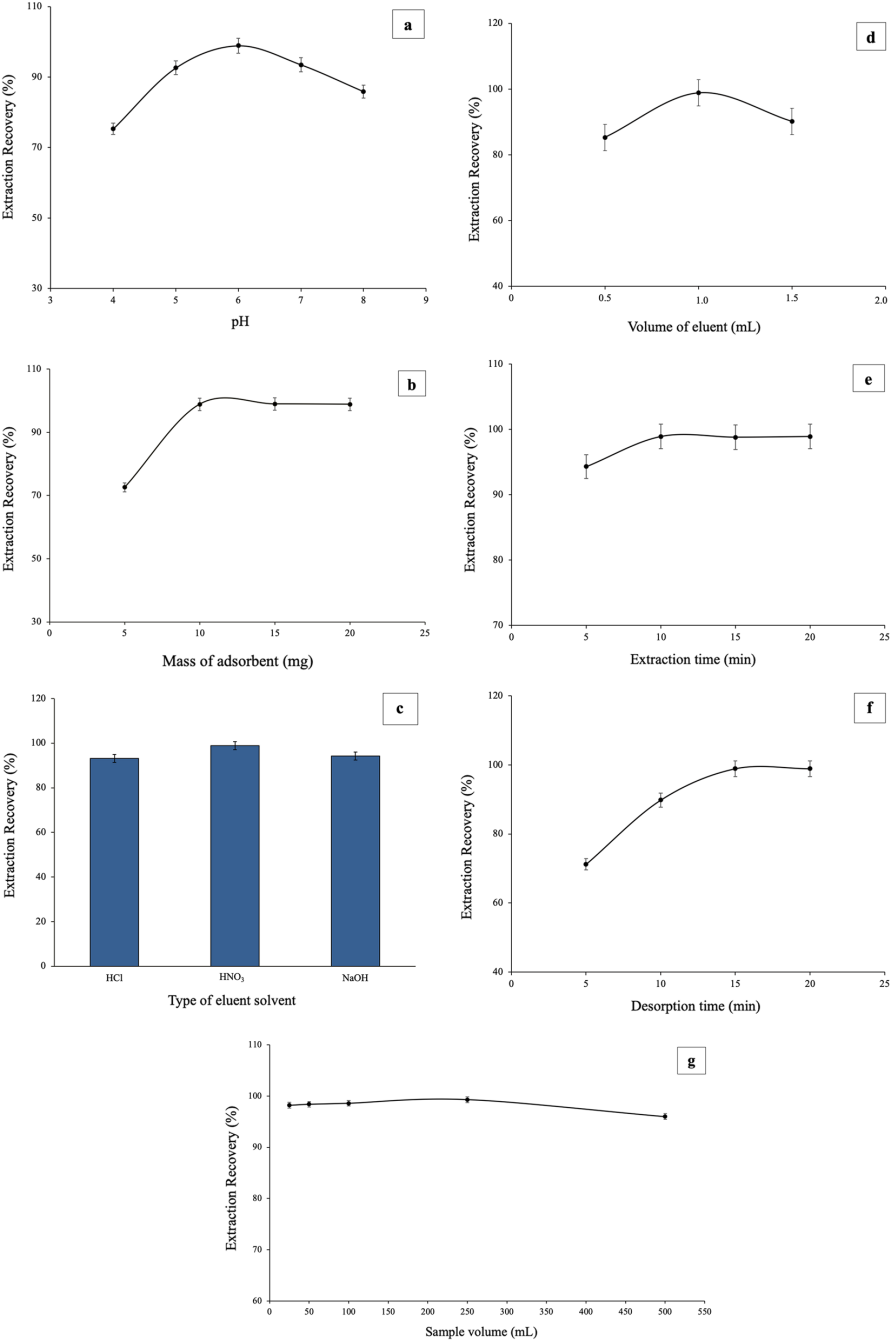
Effect of parameters affecting extraction recovery. pH (a); amount of adsorbent (b); eluent type (c); volume of the eluent (d); extraction time (e); desorption times (f); sample volume (g). In all experiments, 25.0 mL of 100.0 mg L^− 1^ of uranyl ion standard solution was employed and during each experiment, only one parameter at a time was change for optimization

One of the most important parameters affecting SPE is the pH of the sample solution; which besides other effects, has a direct influence on the surface charge of the adsorbent. In this study, the pH of the standard solution was changed in the range of 4.0–8.0 (Fig. 9a). Extraction recovery was increased with increasing pH of solution from 4.0 to 6.0 and then decreased. In pH 6.0, uranium is mostly in the forms of (UO_2_)_2_(OH)_2_^+^, UO_2_OH^+^, and UO_2_^2+^ in solution [33]; and because the isoelectric point of the INA@MOF-808 is around 5.8, the adsorbent has great coordination tendency for these species. Thus, the maximum extraction recovery (ER%) of uranium ions is reached at pH 6.0 which was used as optimum pH in the next experiments.

### Effect of the amount of adsorbent

By varying the amount of the sorbent, its effect on the absorption of the target analyte was investigated. The amount of adsorbent was varied in the range of 5.0–20.0 mg where other extraction parameters were kept constant. The maximum extraction recovery was obtained when the amount of the adsorbent was 10.0 mg and with further increase, ER% remained constant (Fig. 9b). That’s because by increasing the amount of the adsorbent, the accessible active sites to uranyl ions were increased, but after 10.0 mg, almost all analyte ions were already adsorbed by the INA@MOF-808 and absorption signal becomes constant. So, 10.0 mg of adsorbent was chosen as optimum amount in the next experiments.

### Effect of type and volume of the eluent

The Effect of eluent type on the recovery of uranyl ions from the adsorbent was also investigated. To efficiency elute analytes from the sorbent, it is important to choose a sufficient, but as small as possible volume of eluting solvent [34]. To investigate the type of the eluent, 1.0 mL of 1.0 M solution of nitric acid, hydrochloric acid and sodium hydroxide were examined, among them, HNO_3_ showed the highest extraction recovery (Fig. 9c) and was selected for elusion. The effect of the volume of eluting solvent in the range of 0.5 to 1.5 mL was investigated. As shown in Fig. 9d, at the volume of 1.0 mL the maximum recovery of the analyte was achieved. Due to the dilution of eluted uranyl ions, a reverse effect occurs at higher volumes.

### Effect of extraction time

The effect of extraction time was investigated on the extraction recovery in the range of 5.0 to 20.0 min. As can be seen in Fig. 9e, the extraction recovery was increased up to 10.0 min and then remained constant. So, 10.0 min was considered as optimum extraction time.

### Effect of desorption time

Another important parameter which can influence extraction recovery is desorption time of uranyl ions from the adsorbent. Desorption time was investigated in the range of 5.0–20.0 min (Fig. 9f). The best extraction recovery was achieved in 15 min. Therefore, this time was selected as the optimum time for the desorption process and complete elusion of U^(VI)^.

### Effect of sample volume

In all solid phase extraction methods, the volume of sample solution is an important parameter which needs to be optimized to obtain a high preconcentration factor. This effect was studied by varying the sample volume from 25.0 to 500.0 mL, each containing 10 mg of analyte. It was observed that the extraction recovery was increased up to 250.0 mL and then became constant (Fig. 9g). By increasing the volume of sample solution, more analyte can adsorb on the MOF; however, after a certain volume, equilibrium occurs and extraction recovery becomes constant. So, 250.0 mL selected as the optimum volume of the sample solution.

### Effect of interfering ions

Method selectivity toward the target analyte is an important factor for the selective extraction of any ion from the sample solution which needs to be carefully examined. In this study, the selectivity of the synthesized adsorbent on the extraction recovery of uranyl ions was investigated by analyzing standard solutions containing 1.0 mg L^− 1^ of the analyte in the presence of several foreign ions which potentially may coexist with uranium ions in wastewater samples. The tolerance limit was defined as the maximum concentration of the interfering ion causing an error less than ± 5% in the extraction recovery. Results, which are depicted in Table 1, show that even in the presence of the high concentrations of interfering ions, the INA@MOF-808 adsorbent has the capability of selective extraction of the analyte and these ions do not interfere on separation and preconcentration of uranyl ions.

**Table 1:** Effect of interfering ions on the tolerance limits of uranyl ion determination

Ion	Tolerance limit (mg L^− 1^)
Li^+^, Na^+^, K^+^	100
Ca^2+^, Mg^2+^, Ba^2+^	100
Ag^+^, Zn^2+^, Hg^2+^, Co^2+^, Mn^2+^, Cu^2+^, Fe^2+^, Pb^2+^, Sn^2+^, Cr^3+^	10

## Analytical figures of merit

Under the optimum conditions, linearity, detection limit, enrichment factor and precision of the developed method were obtained and summarized in Table 2. Calibration curve was linear in the range of 0.07 µg L^− 1^ to 1.00 mg L^− 1^ with determination coefficient (R^2^) of 0.9986. Based on LOD = 3S_b_/m formula, limit of detection of the method (LOD) calculated as 0.9 µg L^− 1^ (S_b_ is the standard deviation of signals of 10 successive measurements of the blank and *m* is the slope of the calibration curve). By twelve replicate measurements of a 100 mg L^− 1^ standard uranyl solution, precision (as relative standard deviation, RSD%) and inter-day repeatability was calculated and found to be 2.1 and 1.8%, respectively. The enrichment factor (EF) was obtained by using the equation EF = V_i_/V_e_; where V_i_ is the sample volume (mL) and V_e_ is the eluent volume (mL).

**Table 2:** Analytical figure of merit for SPE of uranyl ions using INA@MOF-808 as sorbent

Parameter	Analytical feature
Equation of calibration curve	A = 0.0005C_U_ + 0.0717
Dynamic range (µg L^− 1^)	0.07–1000.00
R^2^ (determination coefficient)	0.9986
Reproducibility (RSD%, n = 12)	2.1
Repeatability (%)	1.8
Limit of detection (µg L^− 1^)	0.90
Enrichment factor	250
Total extraction time (min)	≤ 15

*A* Absorption (analytical) signal, *C*_*U*_ Uranyl ion concentration

In Table 3, the developed protocol is compared to the other SPE-based methods reported for extraction and determination of uranyl ion. As can be seen, LOD and reproducibility of this developed protocol is comparable or superior to the other similar spectrophotometric-based methods. However, due to the high sensitivity of the instrument, methods employing inductively coupled plasma has lower LODs, but still the dynamic linear range of the spectrophotometric-based methods (including this work) are wider. Simplicity of operation with a traditional spectrophotometer is also an important advantage of this method.

**Table 3:** Comparison of the developed method with other methods for the determination of uranyl ions

Sorbent	Instrument	LOD (µg L^− 1^)	Linear range (µg L^− 1^)	RSD (%)	Refs.
MWCNTs/Cu_2_O-CuO	ICP-MS^d^	0.52	2.5–100	1.9	[35]
Br-PADAP^a^-impregnated MWCNTs^c^	ICP-AES^b^	0.14	0.75–225	3.3	[36]
Eu-MOF	Spectrofluorometer	1.59 mg L^− 1^	22.19–155.34 mg L^− 1^	Not mentioned	[37]
Quercetin modified Fe_3_O_4_ nanoparticles	spectrophotometer	4.6	68–6750	3.2	[38]
INA@MOF-808	spectrophotometer	0.9	0.07–1000	2.1	This work

^a^2-(5-Bromo-2-pyridylazo)-5- (diethylamino) phenol; ^b^Inductively coupled plasma atomic emission spectrometer; ^c^Multiwalled carbon nanotube; ^d^Inductively coupled plasma mass spectrometry; ^e^(3-aminopropyl) triethoxysilane

Synthesized adsorbent could be reused at least for eight times without almost any loss of its extraction power, because the crystallinity of the MOF was reserved during the experimental conditions (Fig. 2).

## Real sample analysis

To study the reliability and applicability of the proposed protocol, it was applied for uranyl ion determination in a lake water taken from Chah-Nimeh (Zabol), a local well water (Zabol) and wastewater samples. Water sampleas were analyzed without pre-treatment and wastewater (taken from the urban sewage of Zabol) was filtered through a filter paper before extraction to be free from suspending particles. No analyte was detected in the samples; therefore, to investigate the effect of sample matrix on the extraction, samples were spiked at three concentration levels of 10, 100 and 800 µg L^− 1^ with U(VI). The results, which are shown in Table 4, indicate good recoveries (96.2–97.7%) for the determination of uranyl ions in real water samples. The reproducibility of the method as RSD% was within 1.8–2.8%. Therefore, this procedure was show the high selectivity and capability of the adsorbent for uranium extraction at trace level in real water samples.

**Table 4:** Determination of uranyl ion in real samples

Sample	Analyte added (µg L^− 1^)	Analyte found (µg L^− 1^)	Recovery (%)	RSD% (n = 3)
Ground water	100	97.5	97.5	2.1
	200	195.3	97.7	2.3
Well water	100	96.7	96.7	2.7
	200	193.6	96.8	2.8
Wastewater	100	96.3	96.3	1.9
	200	192.4	96.2	1.8

## Conclusions

In the present study, a porous Zr-based MOF, INA@MOF-808, was prepared for the first time and applied for selective extraction and determination of uranyl ions from aqueous media. Morphological and structural properties of the adsorbent were assessed by FT-IR, PXRD, SEM/EDX, BET surface area and TGA analyses. Presence of vast number of open active zirconium sites, large number of hydroxyl groups, large porosity, very high surface area, and the suitable pore size of the Zr-MOF made it possible to extract uranyl ions selectively and in ultra-trace concentration in complicated matrices such as wastewater samples. A conventional spectrophotometer was used for quantitative analysis. The linear range of the developed method covers a wide range of concentrations. The adsorbent could be used for at least eight extractions without substantial change in its adsorption power. This method has a good reproducibility (RSD ≤ 2.1%) and very low detection limit (0.9 µg L^− 1^). Moreover, spectrophotometric instrumentations own its merits of simplicity, cheapness, portability and so on. The whole analysis time was less than 15 min. Finally, this procedure was used for the extraction and determination of uranyl ions in water samples and acceptable results were achieved. This protocol is simple, economical and fast, with easy sample preparation and requires only a small amount of reagent; therefore, it can be applied for routine U(VI) analysis.

## Data Availability

The majority of the data used to support the findings of this study are included within the article. Other data are available from the corresponding author upon request.

## Abbreviations

BET: Brunauer-Emmett-Teller
BTC: Benzene-1,3,5-tricarboxylic acid (Trimesic acid)
DFT: Density functional theory
DMF: N,N′-dimethyl formamide
EDX: Energy dispersive X-ray
EF: enrichment factor
ER: extraction recovery
FT-IR: Fourier transformed infrared
LOD: limit of detection
MOFs: metal-organic frameworks
PXRD: Powder X-ray diffraction
RSD: relative standard deviations
SEM: Scanning electron microscopy
SPE: Solid phase extraction
TGA: Thermogravimetric analysis
